# Unnatural Disaster: Human Factors in the Mississippi Floods

**DOI:** 10.1289/ehp.116-a390

**Published:** 2008-09

**Authors:** Harvey Black

It’s happened again. For the third time in 15 years the Mississippi River massively burst its banks this spring, inundating tiny Missouri towns such as Winfield (population 720) and Foley (population 178), causing potentially billions of dollars’ worth of destruction—although the damages are still being assessed—and hiking corn prices to $8.00 a bushel in the wake of the lost crop. On 22 April 2008 scientists with the U.S. Geological Survey (USGS) measured the largest water volume on the lower Mississippi River since 1973, with a flow of 1.8 million ft^3^/sec, enough to fill more than 20 Olympic-size swimming pools in 1 second.

The environmental fallout from the 2008 floods is still being assessed. The danger of floodwater is not simply that its level is too high, says Robert Criss, a geologist at Washington University. “Floodwaters are heavily polluted with sewage and filth, commonly bearing counts of coliforms, fecal *Streptococcus*, and other bacteria of many thousands per deciliter,” he explains. “The waters are laden with contaminants including agrochemicals, oils, detergents, and toxic metals. They are highly turbid, typically bearing particulate loads a hundredfold or more higher than normal river waters.” Those pollutants, contaminants, and sediments can be carried into homes, where the lingering dampness promotes mold growth. Moreover, floodwater pools trapped behind failed levees serve as breeding grounds for mosquitoes, flies, and other disease vectors.

Meanwhile, observers are asking how such devastating floods could have occurred again so soon. The massive flooding is attributed largely to torrential spring rains in the Upper Mississippi Valley, which Paul Rydlund, a supervisory hydrologist with the USGS Missouri Water Science Center, says were even greater than those preceding the record-breaking 1993 Midwest flood. But as heavy as those rains were, the question in the minds of some is whether they were made worse by structures such as levees and other man-made interventions wrought upon the Mississippi River over time.

## The Hand of Man

Changes to the river are hardly a latter-day phenomenon. John Anfinson, a historian with the National Park Service’s Mississippi National River and Recreation Area, notes that the early French explorers of the sixteenth and seventeenth centuries recognized that to capture the economic potential of the Mississippi “you have to harness the river; you have to make it behave.” That harnessing began with the settlement of New Orleans in the early 1700s, where the first levees were thrown up to create a city that actually lies below sea level. Thereafter, Anfinson says, “The levee system started growing upriver and the navigation system [i.e., dredging and channeling endeavors] started growing downriver.”

In the centuries that followed, shipping and agricultural interests pressed Congress to allow alteration of the river. Deepening channels made shipping easier, and construction of levees permitted farming and the establishment of towns and cities along the river’s banks.

Many experts point to levees as a major culprit in flood devastation. Levees narrow the flow of the water, preventing it from spreading out into the floodplain and forcing it to move faster, explains geologist Jeffrey Mount, who directs the Center for Watershed Sciences at the University of California, Davis. As a bolus of floodwater moves down a river, levees can get overwhelmed in their work. “The dark secret that no one wants to share is that there are two kinds of levees: those that have failed and those that will fail,” Mount says. And when that levee fails with a massive wall of water pressing on it, the water rushes with great force onto the land behind the levee. “The power of water is a function of the difference in elevation between the top of the water and the adjacent land,” Mount explains. “So the greater that difference, the more powerful the flow that comes out onto that land in terms of its velocity and its power to erode.”

Wing dams on the river are another factor in exacerbating floods, says Criss. These rock jetties, situated roughly perpendicular to the riverbank, aid shipping by preventing the accumulation of sediment so the river channel stays deeper. Deeper water moves faster, meaning the impact of floods can be greater.

Criss points to another problem: “There are general changes to the land that decrease the permeability of soils. As we convert our forests and prairies and fields to subdivisions, we increase the rate of runoff [into the river]—that’s the ubiquitous footprint of man.” According to geologist Nicholas Pinter of Southern Illinois University Carbondale, one particular land use change is quite serious—tile drainage. Because many agricultural soils are too poorly drained to naturally serve as good farmland, farmers install subsurface drains (“tiles”). “When it rains,” Pinter says, “the water comes out of farm fields faster than it would otherwise.”

## Ripe for Disaster?

Land development in floodplains is a perilous exercise, according to Gerald Galloway, a professor of engineering at the University of Maryland and former commander of the U.S. Army Corps of Engineers (USACE). For one thing, he says, developers rely heavily on floodplain maps issued by the Federal Emergency Management Agency (FEMA) that demarcate where we can expect so-called 100-year floods—the sort of flood that can be expected once every 100 years. People erroneously believe if they are outside that demarcation they are safe. However, Galloway cautions, such maps are estimates only. Moreover, he says, “The records of the past don’t necessarily match the pattern we’re in right now in terms of the weather.”

Criss agrees, saying that the rivers of today are not the rivers of the past, and that there is simply not enough historical information on which to base the notion of a 100-or 500-year flood. Besides, at many places, he argues that what have been called 100-year floods are now 10-year floods due to a combination of changing conditions over time.

Larry Buss, who chairs the USACE National Nonstructural/Flood Proofing Committee, says that when a levee is built in compliance with the requirements of the National Flood Insurance Program, there is no longer any floodplain management in the area behind the levee. All kinds of new development can then occur in the area landward of the levee as if the floodplain no longer exists—when in fact it does.

“Politically, it’s more acceptable if politicians come to people and say ‘I’m going to remove the flood threat from you by supporting building a levee so you can live in that floodplain as if the floodplain no longer existed even though flood risk remains,’” Buss says. “That’s more politically acceptable than telling them ‘I’m supporting a buyout’ or ‘I’m supporting a relocation plan where we’ll move you to high ground and have you safe from floods forever.’” However, when the levee does have a problem, the damages are much greater than they might have been because of the increased development in vulnerable areas.

Buss says the U.S. approach to levees has been too focused on what he calls short-term economic/political gain that is ultimately transformed into long-term economic/political loss when major flooding results in levee failure or overtopping and then catastrophic flood damages and perhaps loss of life. In these cases, he says, there is a major disconnect between those who make land use decisions (i.e., local communities) and those who pay for the ill-starred consequences of those decisions (i.e., state and federal taxpayers).

## The Role of the Corps

Noting the impact of man-made modifications of the river, critics question the judgment of the USACE in carrying out such modifications. “The Corps comes in with a community-by-community project with measures like levees, and it looks at that levee; it doesn’t look at the whole Mississippi as a system. And that’s a problem,” says Larry Larson, executive director of the Association of State Floodplain Managers.

Pinter charges that the USACE has failed to examine the possibility of elevated flood risk in many navigational engineering projects, including a $5.3 million project in 2007 to construct three arc-shaped chevrons and other structures in the St. Louis Harbor area. The chevrons work much like wing dams, but they are located wholly in the river rather than extending from the shore. Composed of rock, they are designed to lessen the need for continual dredging of St. Louis Harbor, deepen the river bottom, and straighten the flow of the river. “The benefit of those structures was calculated versus their cost,” says Pinter.

“The benefit was reduced dredging to help maintain the navigation channel. The cost was the construction cost with no calculation whatsoever of the additional financial cost in terms of elevated flood risk.”

But Robert Davinroy, chief of river engineering at the Corps’ St. Louis office, says the USACE did examine the question of elevated flood risk. “We know how these structures work,” he says. “They’re submerged by about twelve feet when the river gets to flood stage—they have no effect on flood heights.” He adds there are no data to show the chevrons have any effect on the height of floods.

As far as levees go, Galloway argues that the USACE is quite interested in understanding the impact of these structures on floods. “In the greater St. Louis area the Corps has been asking [Congress] for money for a study to see what is the cumulative effect of a lot of little levees. So far they haven’t gotten the money. People are not as much interested in learning that sort of information as they are in building levees,” says Galloway, referring to those legislators and constituents who view levees as flood protection. For example, a 20 September 2007 article in *Time* magazine that sharply criticizes a USACE flood control project in Missouri notes that Senator Kit Bond (R–MO) and Representative Jo Ann Emerson (R–MO) were responsible for pushing the project through.

Politics and pressure do play significant roles in the selection of which projects to fund, notes Representative Eddie Bernice Johnson (D–TX), who chairs the Water Resources and Environment Subcommittee of the House Transportation and Infrastructure Committee. For instance, she says the USACE might initially say that a given project is not necessary. “The next time around they’ve been under so much pressure, they’ll go along with it. I’ve seen that happen,” she asserts. She says the pressure comes from local citizens through their representatives for projects that “shouldn’t be funded,” and the prospects for changing the system are dim.

Moreover, Galloway observes that when Congress authorizes a USACE project, the money for it is quickly appropriated. But when it comes to appropriating money to remedy any environmental problems resulting from the project, Congress is very slow to allocate funds, an observation Johnson says is “probably true.”

A 2005 Government Accountability Office report titled *Wetlands Protection: Corps of Engineers Does Not Have an Effective Oversight Approach to Ensure That Compensatory Mitigation Is Occurring* took the Corps’ mitigation efforts to task when it comes to restoring wetlands as part of mitigation efforts, labeling USACE guidelines as “vague and internally inconsistent.” However, by the time the same office issued the 2008 report *Compensatory Mitigation for Losses of Aquatic Resources*, mitigation efforts had reportedly improved and the USACE was meeting mitigation requirements except for the stipulation that any concern voiced about a project receive a 60-day review.

## A New Approach to Managing Floods

From the perspective of the USACE, attitudes toward floods have been evolving into what Buss describes as a more “holistic” approach focused on reducing flood risk with the realization that floods will occur. This approach considers all flood risk reduction tools including not just levees but also buyouts and relocations. Buss says many professional flood risk experts believe the nation should consider levees only as a last resort after first considering measures such as buyouts, relocations, elevation, and zoning.

Using floodplains for any development other than farms is simply asking for trouble, asserts Criss. “Floodplain development should be recognized as geologically stupid, economically unwise, environmentally harmful, and pernicious to mankind,” he says.

Agriculture, however, can make valuable use of the floodplain. Farms in the bottom-lands of rivers tend to be quite productive, says Galloway, whereas “when you start farming in hill country, you’re back to the erosion problems we had in the thirties.” However, some farms are located at especially precarious points along the river—for example, in the former channel of the river. He proposes that such farms be bought out and the levees taken down. But that of course depends on whether the owner of the farmland is willing to sell.

Galloway also proposes changing levees that protect farms so they are open to the river at certain times, as a way to reduce flood damage. It’s an idea that Peter Rabbon, program director of the Corps’ National Flood Risk Management Program, says has merit. One way to implement this idea would be to build an overflow system (or “flowage easement”) into a levee, which Rabbon says would allow water to flow onto farmland in a controlled way.

“This idea has been talked about for ages,” says Mount. “The wisest and best use of these floodplains is farms, rather than cities, because during the hydrologic emergencies [floods] you can store water on those farms, creating a modest amount of dislocation rather than catastrophe. A wise society compensates the farmer for saving it billions of dollars in damages,” he says. Flowage easements are gradually being introduced, he says.

One of the most celebrated buyouts recently was that of Valmeyer, Illinois. The community of 900 souls was devastated by the 1993 Mississippi flood, the latest in a series of inundations endured by that community since 1910. The community moved to higher ground nearby, an effort involving 22 government agencies and a cost in the range of $28 million. In the process of rebuilding, the people of Valmeyer incorporated several sustainable design elements into their new town, including energy-efficient construction and passive solar technology. But such efforts, says Buss, demand strong community leadership with long-term vision—something that can be hard to find.

Whether relocations such as Valmeyer’s will be seen as a result of this year’s flood is still uncertain. Money and leadership are both needed. But recent and future legislation may force at least partial change.

Representative James Oberstar (D–MN), who chairs the House Transportation and Infrastructure Committee, notes that the 2007 $ 23 billion Water Resources Development Act “requires that national water resources planning avoid the unwise use of floodplains and flood-prone areas and requires the President to report by 2010 on national vulnerability to flood damages.” The 2007 legislation also addresses the funding of mitigation efforts by stipulating that if mitigation is required for a particular construction project, then it must be carried out before or concurrently with that project.

This year’s proposed water resource legislation also includes a number of other provisions to reduce flood damage, such as creating incentives to limit development in flood-plains, investing in natural buffers such as wetlands, and pursuing technology for improved understanding of flooding threats. “As we move forward with the next [Water Resources Development Act] bill, we will continue to look for ways to better ensure that mitigation is carried out where and when it is required,” Oberstar says.

## Figures and Tables

**Figure:**
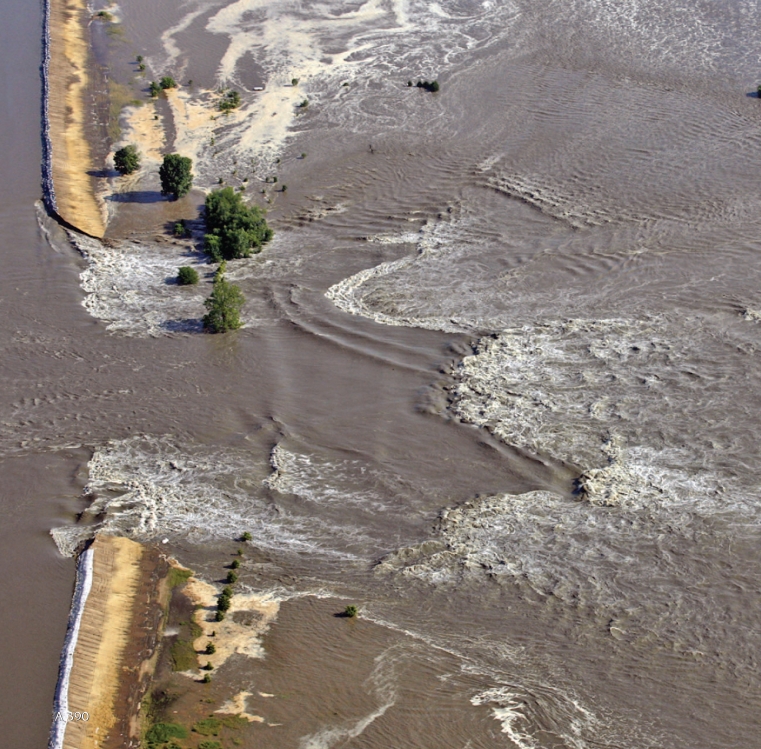
The Mississippi River breaks through a levee between the Illinois towns of Quincy and Meyer, 18 June 2008.

**Figure:**
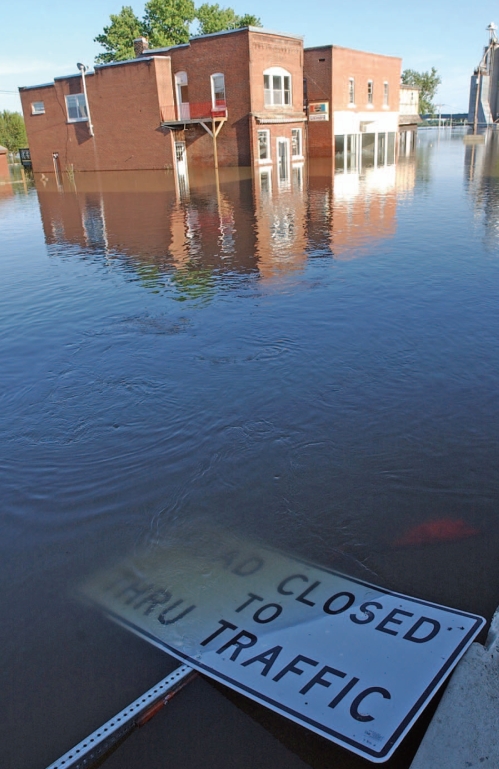
The Mississippi River snakes through downtown LaGrange, Missouri, 21 June 2008. Many engineers and floodplain managers believe the risks that come with siting towns in floodplains are unacceptably high.

